# Pooled extreme-phenotype genome-wide association study XP-GWAS reveals an association between *4-hydroxyphenylpyruvate dioxygenase* and β-carotene variation in *Capsicum annuum*

**DOI:** 10.7717/peerj.21010

**Published:** 2026-03-16

**Authors:** Mesfin Haile, Nayoung Ro, Hyeonseok Oh, Sukyeung Lee, Ho-Cheol Ko, Gi-An Lee

**Affiliations:** 1 National Agrobiodiversity Center, National Institute of Agricultural Sciences, Rural Development Administration (RDA), Jeonju, Jeollabuk-do, Republic of Korea; 2 International Technology Cooperation Center, Rural Development Administration, Jeonju, Jeollabuk-do, Republic of Korea

**Keywords:** β-carotene, *Capsicum annuum*, HPPD, SNP markers, XP-GWAS

## Abstract

**Background:**

Carotenoids are essential plant pigments with key roles in stress tolerance and human nutrition. β-carotene is a major provitamin A carotenoid, and understanding the genetic basis of its natural variation in *Capsicum annuum* is important for nutritional improvement. However, carotenoid accumulation is a complex quantitative trait influenced by multiple metabolic and regulatory pathways.

**Methods:**

An extreme-phenotype genome-wide association study (XP-GWAS) was conducted using pooled genomic DNA from 92 *C. annuum* accessions representing contrasting extremes of β-carotene content. Fruit carotenoid levels from previously characterized accessions were used to establish high- and low-content groups, while genomic DNA from these same accessions was subjected to high-throughput paired-end sequencing. Variant calling yielded 19,066,129 raw variants, which were filtered to 1,025,269 high-confidence single nucleotide polymorphisms (SNPs) for association analysis.

**Results:**

XP-GWAS identified 91 SNPs showing significant allele frequency differences between high- and low–β-carotene pools (FDR < 0.05), with 19 located on assembled chromosomes and 72 on unanchored scaffolds, limiting their immediate utility for functional validation and breeding applications. Among these, 4-hydroxyphenylpyruvate dioxygenase (HPPD) exhibited the most prominent clustered association signal, with multiple significant SNPs overlapping the HPPD gene on chromosome 5. Based on prior studies, HPPD is known to participate in plastoquinone biosynthesis, which indirectly supports carotenoid desaturation; however, the present study identifies a statistical association rather than functional validation in *C. annuum*. Additional SNPs were detected near genes involved in sulfur metabolism, ribosomal function, signaling, and non-coding RNAs, and are interpreted as exploratory, hypothesis-generating signals requiring further validation.

**Conclusions:**

This pooled XP-GWAS prioritizes *HPPD* and several additional genomic regions as candidate loci associated with β-carotene variation in *C. annuum*. Given the exploratory design, pooled sequencing strategy, and prevalence of unanchored scaffold signals, these associations should be viewed as hypothesis-generating and require independent validation before functional or breeding applications.

## Introduction

Carotenoids are a diverse class of isoprenoid pigments synthesized by plants, algae, and certain bacteria. In plants, they perform critical roles in light harvesting, photoprotection, and hormonal regulation, serving as precursors for molecules such as abscisic acid (ABA) and strigolactones (Cazzonelli, 2011; Walter & Strack, 2011). In *Capsicum annuum* (pepper), carotenoids accumulate in fruit pericarp, contributing to the vivid yellow, orange, and red colors that are visually appealing and commercially important (Ha et al., 2007; Guzman et al., 2010).

Among carotenoids, β-carotene is of particular nutritional importance as a major provitamin A compound required for vision, immune function, and normal development in humans (Fraser, 2004; Krinsky & Johnson, 2005). It is abundant in yellow to orange fruits and vegetables such as carrots, mangoes, squash, sweet potatoes, apricots, and dark green leafy vegetables, and is widely distributed in human tissues, particularly in adipose tissue, liver, and muscles (Krinsky & Johnson, 2005; Choi & Yun, 2023). Chemically, β-carotene is a 40-carbon compound containing 15 conjugated double bonds and two β-ionone rings, features that contribute to its hydrophobic nature and stability (Jin & Arroo, 2023). Insufficient β-carotene intake can lead to visual impairment and developmental disorders, whereas adequate consumption has been linked to reduced risks of ophthalmic, metabolic, and cardiovascular diseases (Farasati Far et al., 2023; Bakac et al., 2023).

Genetic mapping studies in *Capsicum* have identified several loci linked to fruit color and pigment composition. Quantitative Trait Locus (QTL) mapping using biparental populations has revealed major-effect loci, particularly those associated with the phytoene synthase 1 (PSY1) and capsanthin-capsorubin synthase (CCS) genes (Lefebvre et al., 1998; Huh et al., 2001). More recently, genome-wide association studies (GWAS) have been used to identify single nucleotide polymorphisms (SNPs) associated with carotenoid-related traits, offering higher resolution than traditional QTL mapping (Ikeogu et al., 2019; Wang et al., 2022; Haile et al., 2023; Chen et al., 2024; Ro et al., 2024). These studies have revealed that, beyond structural biosynthetic enzymes, regulatory factors such as epigenetic modifiers can influence carotenoid accumulation, including a reported association with a histone–lysine N-methyltransferase in *Capsicum* (Haile et al., 2023). Similar regulatory mechanisms have been described in *Citrus* and *Arabidopsis*, suggesting partial conservation of chromatin-mediated control across species (Cazzonelli et al., 2009; Fu et al., 2024).

Despite these advances, carotenoid biosynthesis is not governed solely by variation in core pathway enzymes. Instead, pathway flux is constrained by plastid development, redox balance, and the availability of essential metabolic cofactors. Plastoquinone, for example, serves as a critical redox cofactor for phytoene desaturase, linking carotenoid biosynthesis to upstream metabolic processes such as tyrosine degradation and tocopherol biosynthesis (Norris, Shen & Della Penna, 1998; DellaPenna & Pogson, 2006). Experimental studies in Arabidopsis, tomato, and sweet potato have demonstrated that perturbations in plastoquinone-related metabolism can substantially alter carotenoid accumulation and chromoplast differentiation, highlighting the potential importance of auxiliary metabolic pathways (Norris, Shen & Della Penna, 1998; Kim et al., 2021). However, the contribution of such auxiliary metabolic pathways to natural variation in carotenoid content has not been explored.

Genome-wide association studies offer a powerful framework for identifying loci associated with complex traits, but conventional GWAS often require large sample sizes and may lack power to detect moderate-effect or rare alleles, particularly for environmentally influenced metabolic traits (Korte & Farlow, 2013; Crouch & Bodmer, 2020). Extreme phenotype genome-wide association studies (XP-GWAS) have emerged as an alternative, exploratory strategy for identifying trait-associated loci when sample size is limited (Yang et al., 2015; Gangurde et al., 2022). By sequencing pooled DNA from individuals at phenotypic extremes, XP-GWAS enhances allele frequency contrast, facilitating detection of variants with large allele-frequency differences in moderately sized populations (Yang et al., 2015). However, this design does not capture variants with intermediate or context-dependent effects and limits estimation of individual genotype contributions. Accordingly, XP-GWAS is best suited for prioritizing candidate loci for subsequent validation rather than establishing definitive causal relationships.

In this study, we applied an XP-GWAS approach to explore possible genomic regions associated with β-carotene variation in *C. annuum*. Using pooled whole-genome resequencing of accessions representing extreme β-carotene phenotypes, we aimed to discover candidate SNPs and genes, including those outside the core carotenoid biosynthetic pathway, that may contribute to natural variation in carotenoid accumulation. The results are intended to provide a foundation for future functional validation and evaluation across diverse genetic backgrounds rather than definitive evidence of causal mechanisms.

## Materials and Methods

### Plant materials and carotenoid analysis

A total of 92 *C. annuum* accessions were selected from the National Agrobiodiversity Center germplasm collection based on previously quantified β-carotene content reported by Moon et al. (2023). Carotenoid analysis was performed as described in that study. Freeze-dried and powdered pepper samples were saponified using 3% pyrogallol and 60% potassium hydroxide at 70 °C, followed by extraction with a hexane and ethyl acetate mixture. The extracts were concentrated under nitrogen, resuspended in ethanol, and filtered through a 0.2 µm syringe filter before HPLC analysis. Carotenoids were separated using an Agilent 1260 Infinity II HPLC system (Agilent Technologies, Santa Clara, CA, USA) equipped with a YMC Carotenoid C30 column (250 × 4.6 mm, 5 µm; YMC Co., Ltd., Japan) and detected with a photodiode array at 450 nm. β-carotene content was quantified using external calibration curves generated from β-carotene standard solutions (Sigma-Aldrich, St. Louis, MO, USA) and expressed as µg/g fresh weight following the conversion procedures described by Moon et al. (2023).

No additional carotenoid quantification was performed in the present study. All accessions in the referenced work were analyzed using the same analytical instrumentation, calibration procedures, and reference standards, and the resulting dataset was therefore treated as a consistent phenotypic resource for the present analysis. Nevertheless, the use of previously generated phenotypic data represents an inherent limitation, as environmental and experimental factors associated with the original measurements cannot be fully controlled within the scope of this study. Details on accession identifiers, geographic origins, and β-carotene values are provided in Table S1.

Accessions were classified into two contrasting phenotypic groups representing extremes of β-carotene accumulation. Accessions with β-carotene content ≤ 69.97 µg/g fresh weight were assigned to the low-content group (*n* = 46), while those with β-carotene content ≥ 75.39 µg/g fresh weight were assigned to the high-content group (*n* = 46). This grouping strategy created a clear phenotypic contrast, thereby maximizing allele frequency differences for extreme-phenotype association analysis.

### DNA extraction, DNA pooling and sequencing

Genomic DNA was extracted from young leaf tissue of each selected accession using a modified CTAB method (Lee et al., 2016). Equal amounts of DNA from each accession were combined to form two bulked DNA pools corresponding to the high and low carotenoid groups. DNA quality and concentration were assessed using a Bioanalyzer (Agilent Technologies, Santa Clara, CA, USA) and qPCR. Library preparation was performed using the Illumina TruSeq DNA PCR-Free Library Prep Kit, and fragment sizes and concentrations were evaluated with Picogreen assays and qPCR. Libraries passed quality control thresholds and were sequenced on an Illumina NovaSeq 6000 platform, generating 150 bp paired-end reads.

Raw sequencing reads were quality-checked using FastQC (Andrews, 2010), and adapter trimming as well as quality filtering were conducted with Trimmomatic v0.39 (Bolger, Lohse & Usadel, 2014). Filtered reads were aligned to the *C. annuum* reference genome (https://www.ncbi.nlm.nih.gov/datasets/genome/GCF_002878395.1/) using BWA-MEM v0.7.17 (Li & Durbin, 2009).

### SNP calling and filtering

Aligned reads were processed with Picard v2.26.4 (Broad Institute, 2019) to assign read groups and mark duplicates. Indel realignment was performed using GATK v4.2.0.0 (McKenna et al., 2010), followed by SNP calling with GATK’s HaplotypeCaller. Variant filtering was carried out with VCFtools v0.1.16 (Danecek et al., 2011).

To improve the reliability of allele frequency estimation in pooled sequencing data, SNPs were filtered based on sequencing depth. Variants were retained if they had a minimum reference or alternate allele depth of ≥50 reads in at least one pooled sample, ensuring sufficient read support for robust allele frequency comparisons between the high- and low-β-carotene pools. After filtering, 1,025,269 high-confidence SNPs were retained for downstream analyses.

### XP-GWAS analysis, SNP annotation and candidate identification

XP-GWAS was conducted using a modified R script adapted from the XP-GWAS pipeline (https://github.com/schnablelab/XP-GWAS) (Yang et al., 2015). For each SNP, allele frequency differences between the high- and low-carotenoid pools were tested, and *p*-values were computed. Association testing employed a general linear model (GLM), and quantile–quantile (QQ) plots were generated to assess model accuracy (Fig. S1). Genomic inflation was assessed using the inflation factor (*λ*), and multiple testing correction was applied using the False Discovery Rate (FDR) method (Benjamini & Hochberg, 1995). SNPs with FDR <0.05 were considered statistically significant.

Functional annotation of significant SNPs was performed using SnpEff v4.3t (Cingolani et al., 2012), predicting variant impacts and associating SNPs with gene models based on the *C. annuum* reference genome. Genes were considered associated only when the significant SNP physically resided within an annotated gene region (coding sequence, intron, or untranslated region). SNPs located in intergenic regions were not assigned to candidate genes; instead, the nearest upstream and downstream annotated genes were reported solely to provide genomic context without implying functional association.

## Results

### β-carotene content in high and low accession groups

**Figure 1:**
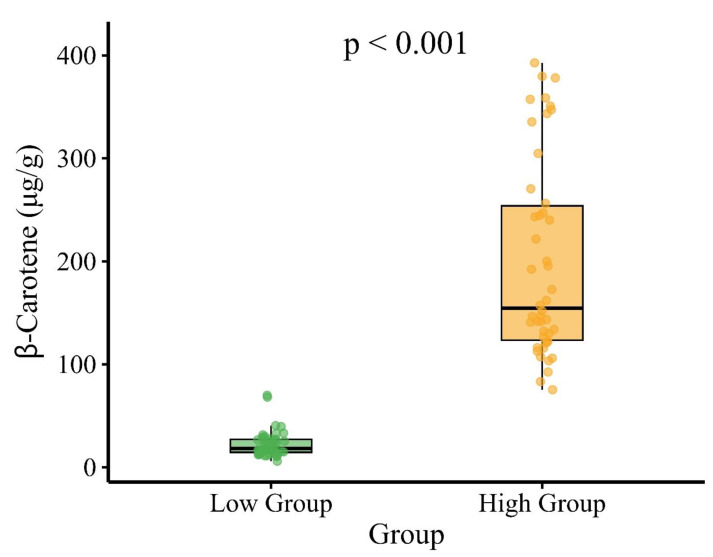
Boxplot of β-carotene content (μg/g) in two groups (“High” and “Low”). Individual data points are shown with jittered scatter to illustrate distribution. Colors indicate group membership (yellow for High, green for Low). The Wilcoxon rank-sum test was performed to compare β-carotene levels between groups, with the resulting *p*-value displayed above the plot.

As expected from the phenotype-based selection, β-carotene content differed significantly between the high- and low-carotenoid groups. The high group exhibited a mean concentration of 199.24 μg/g (range: 75.39 to 392.74 *μ*g/g) with greater variability (standard deviation = 95.94 *μ*g/g), while the low group averaged 22.53 μg/g (range: 5.97 to 69.97 μg/g) with less variation (standard deviation = 12.81 μg/g) (Table 1). Median values were consistent with these findings, at 154.59 μg/g and 18.3 μg/g for the high and low groups, respectively. These differences are illustrated in Fig. 1, which presents a boxplot of β-carotene content by group. Individual accessions are represented by jittered points colored by group, revealing the greater spread in the high group compared to the low group. A Wilcoxon rank-sum test confirmed the difference between groups to be statistically significant (*p* < 0.001), with the *p*-value displayed above the plot. This clear separation validates the grouping strategy and establishes a robust basis for subsequent genetic analyses.

**Table 1:** Summary statistics of β-carotene content in high and low carotenoid accession groups.

**Statistic**	**High group**	**Low group**
Mean (μg/g)	199.24	22.53
Median (μg/g)	154.59	18.3
Standard deviation	95.94	12.81
Range (μg/g)	317.35	64
Minimum (μg/g)	75.39	5.97
Maximum (μg/g)	392.74	69.97
Sample size (n)	46	46
Confidence interval (95%)	±28.49	±3.81

### Sequencing and alignment results

High-throughput sequencing of the pooled DNA samples yielded high-quality data for both groups (Table 2). The high β-carotene pepper pool produced 481.97 million raw reads, with 99.83% of paired reads retained after trimming and only 0.05% discarded. Similarly, the low β-carotene pepper pool generated 498.69 million raw reads, with 99.85% retention and minimal read loss. Trimmed reads were mapped to the *C. annuum* reference genome with high efficiency. Mapping rates exceeded 99.3% for both pools, with 956.1 million and 989.6 million reads successfully aligned in the high and low β-carotene pools, respectively (Table 3). Average sequencing depths were 44.1 × for the high β-carotene pool and 45.7 × for the low β-carotene pool, ensuring sufficient coverage for reliable variant detection and association analysis.

**Table 2:** Raw sequencing and trimming summary for pepper samples.

No	β-carotene group	Raw reads			Trimmed base pairs (bp)		
		Total	bp	Read length (bp)	Total paired	Both surviving	Dropped
1	High	2 × 48,1967,099	2 × 7277,7031,949	151	481,967,099	481,153,663 (99.83%)	248,261 (0.05%)
2	Low	2 × 49,8685,327	2 × 7530,1484,377	151	498,685,327	497,949,532 (99.85%)	229,319 (0.05%)

**Table 3:** Read alignment and mapping statistics for pepper samples.

No	Sample ID	Reads aligned				
		Total reads	Mapped reads	Unmapped reads	Mapping rates	Depth
1	High β-carotene group	962,307,326	956,118,031	6,189,295	99.36%	44.1
2	Low β-carotene group	995,899,064	989,646,002	6,253,062	99.37%	45.7

### Variant calling and filtering

A total of 19,066,129 raw variants were initially identified from the aligned sequencing data. Following allele frequency estimation, quality control procedures were applied to retain only high-confidence SNPs. Variants with reference or alternate allele depth below 50 in the pooled samples were excluded, resulting in a final dataset of 1,025,269 high-quality SNPs used for downstream analyses.

**Figure 2:**
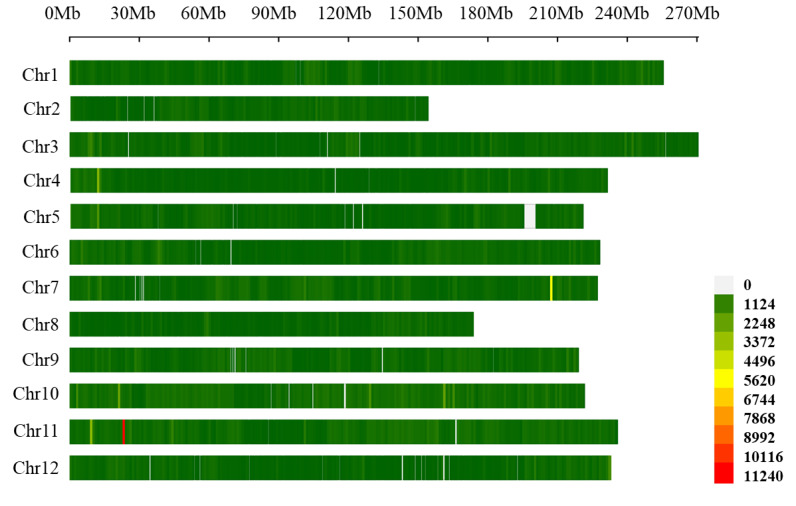
SNP density was measured for each chromosome region using the number of SNPs within a 1 Mb sliding window. The plot represents the variation in SNP density across the different chromosomes.

The distribution of SNPs across the *C. annuum* genome was generally uniform, although differences in density were observed among chromosomes. Chromosome 10 contained the highest number of SNPs (91,418), whereas Chromosome 8 exhibited the lowest density (25,427) (Table 4). A genome-wide SNP density heatmap was generated to visualize these patterns. Using a 1 Mb sliding window, the plot revealed distinct regions with elevated or reduced polymorphism, which may correspond to genomic regions under selection or with low genetic diversity (Fig. 2).

**Table 4:** Summary of the number of final SNP loci identified per chromosome.

Chromosome	SNPs	Chromosome	SNPs
Chr1	54,228	Chr7	60,801
Chr2	31,643	Chr8	25,427
Chr3	53,198	Chr9	56,101
Chr4	35,352	Chr10	91,418
Chr5	44,516	Chr11	87,660
Chr6	48,139	Chr12	38,206

#### XP-GWAS identifies significant SNPs

**Figure 3:**
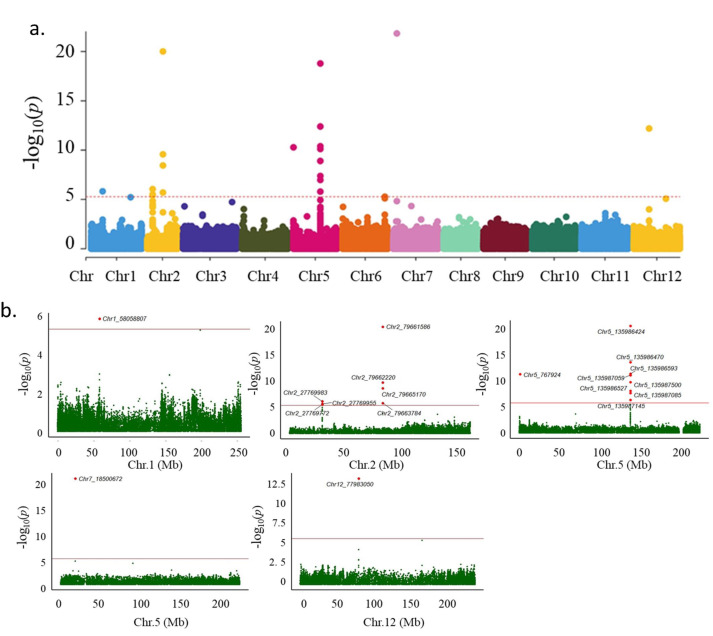
Genome-wide association analysis of β-carotene content in *C. annuum*. (A) Manhattan plot showing −log_10_ *p*-values for SNPs across all 12 chromosomes, colored by chromosome. (B) Chromosome-wise Manhattan plots. The red line marks the genome-wide significance threshold (FDR-adjusted *p* < 0.05). Significant peaks appear on chromosomes 2, 5, 7, and 12. The *x*-axis represents physical position (Mb), and the *y*-axis shows −log_10_ *p*-values. Lead SNPs are highlighted in red. These regions include candidate genes involved in carotenoid biosynthesis, plastid development, or regulatory functions.

XP-GWAS analysis was performed on 1,025,269 high-confidence SNPs to identify genomic regions associated with carotenoid content variation between the high- and low-carotenoid pools. The genomic inflation factor (*λ*) approximated 1, indicating minimal genomic inflation and supporting the statistical robustness of the analysis. After multiple testing correction using the False Discovery Rate (FDR) method, 91 SNPs were deemed significantly associated with carotenoid levels (FDR <0.05). Among these, 19 SNPs mapped to assembled chromosomes (Table 5), while 72 were located on unanchored scaffolds. A notable cluster of significant SNPs was identified within the 4-hydroxyphenylpyruvate dioxygenase (HPPD; LOC107870882) gene on chromosome 5 (Table 5). This gene is implicated in plastoquinone biosynthesis, a critical component for carotenoid biosynthesis, suggesting a plausible functional role in carotenoid accumulation in *C. annuum*. Additional genomic signals were observed on chromosomes 1, 2, 5, 7, and 12 (Fig. 3).

**Table 5:** Top SNPs significantly associated with β-carotene content in *C. annuum*based on XP-GWAS analysis.

No.	Marker	Chr.	Pos.	*q*-value	SNP type	Gene
1	Chr1_58058807	Chr1	5,8058,807	1.93 × 10^−2^	Intergenic	uncharacterized/rho GTPase-activating protein 7
2	Chr2_79661586	Chr2	7,9661,586	5.96 × 10^−16^	Intergenic	adenylyl-sulfate kinase / 60S ribosomal protein L26-1
3	Chr2_79662220	Chr2	7,9662,220	6.29 × 10^−6^	Intergenic	adenylyl-sulfate kinase/60S ribosomal protein L26-1
4	Chr2_79665170	Chr2	7,9665,170	7.77 × 10^−5^	Intergenic	adenylyl-sulfate kinase/60S ribosomal protein L26-1
5	Chr2_27769983	Chr2	2,7769,983	1.26 × 10^−2^	Intron variant	ribosome biogenesis protein NOP53
6	Chr2_79663784	Chr2	7,9663,784	2.50 × 10^−2^	Intron variant	adenylyl-sulfate kinase / 60S ribosomal protein L26-1
7	Chr2_27769955	Chr2	2,7769,955	3.71 × 10^−2^	Intron variant	ribosome biogenesis protein NOP53
8	Chr2_27769772	Chr2	2,7769,772	4.43 × 10^−2^	Intron variant	ribosome biogenesis protein NOP53
9	Chr5_135986424	Chr5	13,5986,424	9.38 × 10^−15^	Intron variant	4-hydroxyphenylpyruvate dioxygenase
10	Chr5_135986470	Chr5	13,5986,470	1.66 × 10^−8^	Intron variant	4-hydroxyphenylpyruvate dioxygenase
11	Chr5_135986593	Chr5	13,598,6593	1.20 × 10^−6^	Intron variant	4-hydroxyphenylpyruvate dioxygenase
12	Chr5_135987059	Chr5	13,5987,059	1.99 × 10^−6^	Intron variant	4-hydroxyphenylpyruvate dioxygenase
13	Chr5_135987500	Chr5	13,5987,500	2.90 × 10^−5^	Intron variant	4-hydroxyphenylpyruvate dioxygenase
14	Chr5_135986527	Chr5	13,5986,527	7.15 × 10^−4^	Intron variant	4-hydroxyphenylpyruvate dioxygenase
15	Chr5_135987085	Chr5	13,5987,085	1.69 × 10^−3^	Intron variant	4-hydroxyphenylpyruvate dioxygenase
16	Chr5_135987145	Chr5	13,5987,145	2.02 × 10^−2^	Intron variant	4-hydroxyphenylpyruvate dioxygenase
17	Chr5_767924	Chr5	767,924	1.41 × 10^−6^	Intron variant	pentatricopeptide repeat-containing protein
18	Chr7_18500672	Chr7	1,8500,672	1.02 × 10^−17^	Intron variant	5S ribosomal RNA
19	Chr12_77983050	Chr12	7,7983,050	2.38 × 10^−8^	Intergenic	small nucleolar RNA R71/no_match

Significant associations were also identified near genes involved in sulfur metabolism, ribosome biogenesis, and signal transduction. These include SNPs in or near adenylyl-sulfate kinase, 60S ribosomal protein L26-1, ribosome biogenesis protein NOP53, and Rho GTPase-activating protein 7. Although these genes are not directly linked to carotenoid biosynthesis, their proximity to significant SNPs suggests a possible indirect contribution through broader metabolic or regulatory pathways. In addition, several associated variants were found near non-coding RNAs such as 5S ribosomal RNA and small nucleolar RNA R71, indicating potential regulatory influences on pathways related to carotenoid accumulation. Further experimental validation is required to determine their functional relevance.

**Figure 4:**
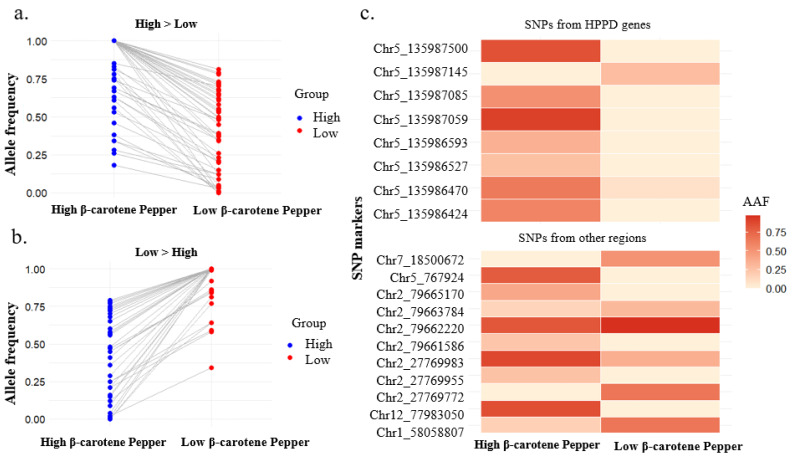
Allelic frequency analysis of significant SNPs associated with β-carotene content in *C. annuum*. (A, B) Allelic frequencies of all 91 significant SNPs identified by XP-GWAS, showing the proportion of SNP alleles more frequent in the high-carotenoid group (58%, 53/91) *versus* the low-carotenoid group (42%, 38/91). (C) Heat map of allelic frequencies for significant SNPs specifically located within the HPPD gene and other chromosome regions, excluding scaffold variants. SNPs more frequent in the high β-carotene group are highlighted, supporting a potential role of HPPD variation in carotenoid accumulation.

Allelic frequency analysis of the 91 significant SNPs revealed that 58% (53/91) were more frequent in the high-carotenoid group, whereas 42% (38/91) predominated in the low-carotenoid group (Figs. 4A, 4B). Among these, 19 SNPs were situated within chromosome regions and 72 within scaffold regions. Figures 4A and 4B display the overall allelic frequencies of all significant SNPs, encompassing both chromosome and scaffold locations. Conversely, Fig. 4C presents a heat map focusing exclusively on SNPs from the HPPD gene and other chromosome regions, excluding scaffold-derived variants. Strong association signals were confirmed within the HPPD gene region, where multiple significant SNPs were more commonly observed in the high β-carotene group, suggesting a potential positive association between HPPD variation and carotenoid accumulation. Collectively, these findings provide supportive evidence for a role of HPPD in regulating β-carotene content, consistent with its established function in carotenoid biosynthesis.

## Discussion

This discussion interprets the identified associations within an exploratory XP-GWAS framework. Given that 1,025,269 SNPs were tested using a FDR threshold of 0.05, a proportion of the 91 significant SNPs is statistically expected to represent false-positive associations. Accordingly, the signals discussed below, including those involving *HPPD*, should be considered exploratory insights rather than definitive evidence of causality.

Carotenoid biosynthesis is controlled by a well-defined pathway involving key enzymes such as phytoene synthase (PSY), phytoene desaturase (PDS), *ζ*-carotene desaturase (ZDS), lycopene β-cyclase (LCYB), and β-carotene hydroxylase (CHYB) (Giuliano, 2017; Rodriguez-Concepcion et al., 2018). Both transcriptional and post-transcriptional processes contribute to regulation, and factors like feedback mechanisms, plastid maturation, and precursor supply shape the overall pathway activity (Cazzonelli & Pogson, 2010; Toledo-Ortiz, Huq & Rodríguez-Concepción, 2010; Ruiz-Sola & Rodríguez-Concepción, 2012; Li & Yuan, 2013; Sun et al., 2018).

XP-GWAS identified SNPs within the *HPPD* gene (LOC107870882) on chromosome 5 that were associated with variation in β-carotene content. These associations indicate that *HPPD* represents a candidate locus of interest within the analyzed population, rather than evidence of a definitive or singular genetic determinant. Although *HPPD* is not a structural gene in the carotenoid biosynthetic pathway, it produces homogentisate, a precursor of plastoquinone, which serves as an essential redox cofactor for phytoene desaturase (Norris, Barrette & DellaPenna, 1995; DellaPenna & Pogson, 2006; Rodriguez-Concepcion et al., 2018). This indirect biochemical connection provides a plausible mechanistic basis for the observed association, without implying a direct regulatory role.

Evidence from model and crop species indicates that perturbation of *HPPD* affects plastidial redox metabolism and carotenoid-related phenotypes (Norris, Barrette & DellaPenna, 1995; Kim et al., 2021). However, such findings primarily demonstrate pathway connectivity rather than trait causality, and direct functional evidence linking natural *HPPD* variation to β-carotene accumulation in *C. annuum* is currently unavailable. The identified SNPs include nonsynonymous and intronic variants, which may affect enzyme function or gene regulation, but their phenotypic relevance in pepper remains to be established through replication and functional analyses.

**Figure 5:**
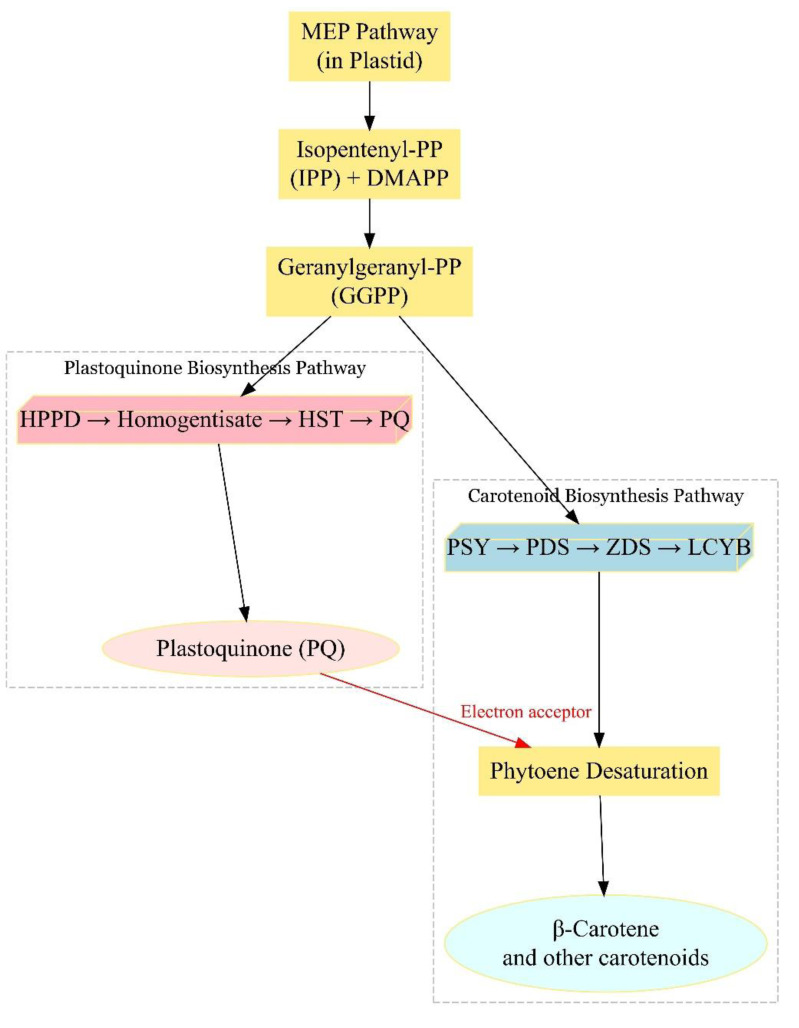
Proposed metabolic relationship between 4-hydroxyphenylpyruvate dioxygenase and β-carotene accumulation in *C. annuum*. The connection among the methylerythritol 4-phosphate isoprenoid pathway, plastoquinone biosynthesis, and carotenoid production within plastids. The methylerythritol 4-phosphate pathway provides the universal five-carbon precursors isopentenyl pyrophosphate and dimethylallyl pyrophosphate, which combine to form geranylgeranyl pyrophosphate, a key substrate shared by both carotenoid and plastoquinone biosynthesis. The gene identified by extreme phenotype genome-wide association study, 4-hydroxyphenylpyruvate dioxygenase, catalyzes the formation of homogentisate, an essential precursor for plastoquinone. Plastoquinone acts as an important electron acceptor for phytoene desaturase and zeta-carotene desaturase, enzymes required to convert colorless phytoene into colored carotenoids. Therefore, HPPD indirectly promotes beta-carotene accumulation by sustaining the plastoquinone pool necessary for these desaturation steps. Arrows indicate the biosynthetic flow and dashed boxes highlight major branches of the pathway.

Importantly, Fig. 5 illustrates the metabolic interplay between the MEP pathway, plastoquinone biosynthesis, and carotenoid production. Carotenoid precursors mainly come from the plastid-localized methylerythritol 4-phosphate (MEP) pathway, which produces the five-carbon units isopentenyl pyrophosphate (IPP) and dimethylallyl pyrophosphate (DMAPP) (Rodríguez-Concepción, 2010). These combine to form geranylgeranyl pyrophosphate (GGPP), a key substrate shared by carotenoid and plastoquinone biosynthesis. Although the cytosolic mevalonate (MVA) pathway also produces IPP, its contribution to carotenoid biosynthesis is generally minor (Rodríguez-Concepción, 2010). This metabolic integration underscores how enzymes outside the canonical carotenoid pathway may influence carotenoid accumulation indirectly through plastidial cofactor supply.

Significant SNPs were distributed across both chromosome-anchored regions and unanchored scaffolds. Of the identified variants, only a subset could be mapped to assembled chromosomes, whereas the majority resided on unanchored scaffolds, reflecting current limitations of the *C. annuum* reference genome rather than an absence of biologically relevant variation. While chromosome-anchored SNPs allow more immediate positional interpretation, scaffold-based associations should be considered provisional until improvements in genome assembly and annotation permit more precise localization and functional assessment.

Significant SNPs were also detected near genes involved in sulfur metabolism, ribosomal function, cellular signaling, and non-coding RNAs. Given the number of variants tested and the statistical properties of FDR-controlled analyses, a proportion of such associations is expected to arise from stochastic allele frequency differences rather than true biological enrichment. Accordingly, these loci should be interpreted cautiously and viewed as exploratory signals rather than confirmed contributors to carotenoid biosynthesis. The potential indirect links discussed, such as redox homeostasis or translational regulation are therefore presented as biologically plausible hypotheses that warrant further investigation rather than mechanistic conclusions.

Possible limitations should be considered when interpreting these results. The XP-GWAS design contrasts pooled extreme phenotypes but does not explicitly control for population stratification or relatedness among accessions. If the high- and low–β-carotene groups differ in genetic ancestry, geographic origin, or breeding background, some allele frequency differences may reflect shared genomic structure rather than loci causally related to carotenoid biosynthesis. In addition, while allele frequency divergence suggests potential for marker development, the identified SNPs should be regarded as preliminary candidates rather than immediately actionable breeding markers. The detected variants likely explain only a fraction of the genetic variance underlying β-carotene content, and many are located on unanchored scaffolds that currently limit assay development and functional interpretation. Consequently, the primary contribution of this study is the prioritization of candidate loci and pathways for further investigation, with independent validation across diverse germplasm and environments required before translation into marker-assisted or genomic selection.

## Conclusion

Using an extreme-phenotype GWAS approach, this study explored genomic regions associated with variation in β-carotene content in *C. annuum*. The analysis identified SNPs associated with the HPPD locus, supporting its role as a biologically plausible candidate gene rather than a confirmed causal factor. These findings extend the genetic landscape of carotenoid variation beyond core biosynthetic genes, suggesting indirect contributions from cofactor-related and auxiliary metabolic pathways. Given the exploratory nature of the pooled XP-GWAS design, the identified associations should be interpreted as hypothesis-generating, providing a foundation for future validation and breeding-oriented investigations aimed at improving nutritional quality in pepper.

## Supplemental Information

10.7717/peerj.21010/supp-1 Supplemental Information 1 Detail information of the 92 pepper accessions

10.7717/peerj.21010/supp-2 Supplemental Information 2 Quantile–Quantile (QQ) plots showing the distribution of observed versus expected test statistics from the XP-GWAS for β- carotenoid content in Capsicum annuum (Left) Before genomic control correction, the inflation factor (*λ*) was 19.94, indicating substantial test statistic inflation. (Right) After (*λ*) correction, the inflation factor was reduced to 1, suggesting effective control for population structure or other confounding factors. Each point represents a single SNP. The solid diagonal line represents the null hypothesis of no association.

## Additional Information and Declarations

### Data Availability

The following information was supplied regarding data availability: The sequence data are available at NCBI SRA: PRJNA1431431.
