# Supplementary material for: Pooled extreme-phenotype genome-wide association study XP-GWAS reveals an association between 4-hydroxyphenylpyruvate dioxygenase and β-carotene variation in Capsicum annuum
Source: PeerJ. 2026 Mar 16;14:e21010. doi: 10.7717/peerj.21010 (PMC13092231; doi:10.7717/peerj.21010)
Supplement: Supplemental Information 1 [file peerj-14-21010-s001.docx]

| No. | IT number | Species name | β-Carotene | Group |
| --- | --- | --- | --- | --- |
| 1 | 311483 | *Capsicum annuum* | 392.74 | High |
| 2 | 311497 | *Capsicum annuum* | 379.71 | High |
| 3 | 311489 | *Capsicum annuum* | 378.15 | High |
| 4 | 330670 | *Capsicum annuum* | 358.72 | High |
| 5 | 311488 | *Capsicum annuum* | 357.35 | High |
| 6 | 311496 | *Capsicum annuum* | 350.77 | High |
| 7 | 311494 | *Capsicum annuum* | 347.23 | High |
| 8 | 311485 | *Capsicum annuum* | 343.54 | High |
| 9 | 311491 | *Capsicum annuum* | 335.5 | High |
| 10 | 311493 | *Capsicum annuum* | 304.83 | High |
| 11 | 229393 | *Capsicum annuum* | 270.49 | High |
| 12 | 330681 | *Capsicum annuum* | 256.26 | High |
| 13 | 311490 | *Capsicum annuum* | 246.93 | High |
| 14 | 330734 | *Capsicum annuum* | 244.58 | High |
| 15 | 324816 | *Capsicum annuum* | 243.33 | High |
| 16 | 330666 | *Capsicum annuum* | 240.09 | High |
| 17 | 330667 | *Capsicum annuum* | 221.69 | High |
| 18 | 330672 | *Capsicum annuum* | 200.19 | High |
| 19 | 330675 | *Capsicum annuum* | 195.56 | High |
| 20 | 293669 | *Capsicum annuum* | 192.35 | High |
| 21 | 270566 | *Capsicum annuum* | 172.7 | High |
| 22 | 330685 | *Capsicum annuum* | 162.05 | High |
| 23 | 324815 | *Capsicum annuum* | 157.32 | High |
| 24 | 330638 | *Capsicum annuum* | 151.85 | High |
| 25 | 330674 | *Capsicum annuum* | 146.71 | High |
| 26 | 330655 | *Capsicum annuum* | 146.56 | High |
| 27 | 330682 | *Capsicum annuum* | 143.49 | High |
| 28 | 330648 | *Capsicum annuum* | 141.88 | High |
| 29 | 330707 | *Capsicum annuum* | 141.56 | High |
| 30 | 330720 | *Capsicum annuum* | 140.86 | High |
| 31 | 228172 | *Capsicum annuum* | 133.89 | High |
| 32 | 311495 | *Capsicum annuum* | 132.06 | High |
| 33 | 311508 | *Capsicum annuum* | 130.08 | High |
| 34 | 330717 | *Capsicum annuum* | 126.57 | High |
| 35 | 330659 | *Capsicum annuum* | 122.41 | High |
| 36 | 330651 | *Capsicum annuum* | 121.8 | High |
| 37 | 311524 | *Capsicum annuum* | 120.6 | High |
| 38 | 100765 | *Capsicum annuum* | 116.18 | High |
| 39 | 330733 | *Capsicum annuum* | 115.78 | High |
| 40 | 324813 | *Capsicum annuum* | 112.77 | High |
| 41 | 330679 | *Capsicum annuum* | 107.37 | High |
| 42 | 311514 | *Capsicum annuum* | 106.02 | High |
| 43 | 158695 | *Capsicum annuum* | 103.46 | High |
| 44 | 240355 | *Capsicum annuum* | 92.55 | High |
| 45 | 330647 | *Capsicum annuum* | 83.25 | High |
| 46 | 330712 | *Capsicum annuum* | 75.39 | High |
| 47 | 290142 | *Capsicum annuum* | 69.97 | Low |
| 48 | 294677 | *Capsicum annuum* | 68.16 | Low |
| 49 | 267543 | *Capsicum annuum* | 40.52 | Low |
| 50 | 311552 | *Capsicum annuum* | 39.60 | Low |
| 51 | 264053 | *Capsicum annuum* | 33.27 | Low |
| 52 | 264056 | *Capsicum annuum* | 33.15 | Low |
| 53 | 270698 | *Capsicum annuum* | 31.53 | Low |
| 54 | 270598 | *Capsicum annuum* | 29.87 | Low |
| 55 | 158764 | *Capsicum annuum* | 29.30 | Low |
| 56 | 267545 | *Capsicum annuum* | 28.22 | Low |
| 57 | 218617 | *Capsicum annuum* | 27.45 | Low |
| 58 | 296555 | *Capsicum annuum* | 27.19 | Low |
| 59 | 203221 | *Capsicum annuum* | 26.96 | Low |
| 60 | 297533 | *Capsicum annuum* | 26.67 | Low |
| 61 | 270436 | *Capsicum annuum* | 25.69 | Low |
| 62 | 294690 | *Capsicum annuum* | 25.43 | Low |
| 63 | 294689 | *Capsicum annuum* | 25.36 | Low |
| 64 | 270766 | *Capsicum annuum* | 22.59 | Low |
| 65 | 294693 | *Capsicum annuum* | 22.26 | Low |
| 66 | 138200 | *Capsicum annuum* | 22.14 | Low |
| 67 | 267516 | *Capsicum annuum* | 21.58 | Low |
| 68 | 231383 | *Capsicum annuum* | 20.07 | Low |
| 69 | 100777 | *Capsicum annuum* | 18.32 | Low |
| 70 | 319741 | *Capsicum annuum* | 18.28 | Low |
| 71 | 294692 | *Capsicum annuum* | 17.83 | Low |
| 72 | 299451 | *Capsicum annuum* | 17.4 | Low |
| 73 | 136646 | *Capsicum annuum* | 17.01 | Low |
| 74 | 158552 | *Capsicum annuum* | 16.83 | Low |
| 75 | 296536 | *Capsicum annuum* | 15.87 | Low |
| 76 | 297530 | *Capsicum annuum* | 15.86 | Low |
| 77 | 296563 | *Capsicum annuum* | 15.3 | Low |
| 78 | 319738 | *Capsicum annuum* | 14.98 | Low |
| 79 | 332712 | *Capsicum annuum* | 14.76 | Low |
| 80 | 296564 | *Capsicum annuum* | 14.62 | Low |
| 81 | 138201 | *Capsicum annuum* | 14.57 | Low |
| 82 | 319740 | *Capsicum annuum* | 13.61 | Low |
| 83 | 319739 | *Capsicum annuum* | 13.33 | Low |
| 84 | 319735 | *Capsicum annuum* | 13.18 | Low |
| 85 | 297531 | *Capsicum annuum* | 12.76 | Low |
| 86 | 284118 | *Capsicum annuum* | 12.47 | Low |
| 87 | 319734 | *Capsicum annuum* | 12.3 | Low |
| 88 | 264054 | *Capsicum annuum* | 11.36 | Low |
| 89 | 297529 | *Capsicum annuum* | 11.3 | Low |
| 90 | 296664 | *Capsicum annuum* | 10.9 | Low |
| 91 | 229396 | *Capsicum annuum* | 10.7 | Low |
| 92 | 286135 | *Capsicum annuum* | 5.97 | Low |
