# Supplementary figures and images for: Pooled extreme-phenotype genome-wide association study XP-GWAS reveals an association between 4-hydroxyphenylpyruvate dioxygenase and β-carotene variation in Capsicum annuum
Source: PeerJ. 2026 Mar 16;14:e21010. doi: 10.7717/peerj.21010 (PMC13092231; doi:10.7717/peerj.21010)

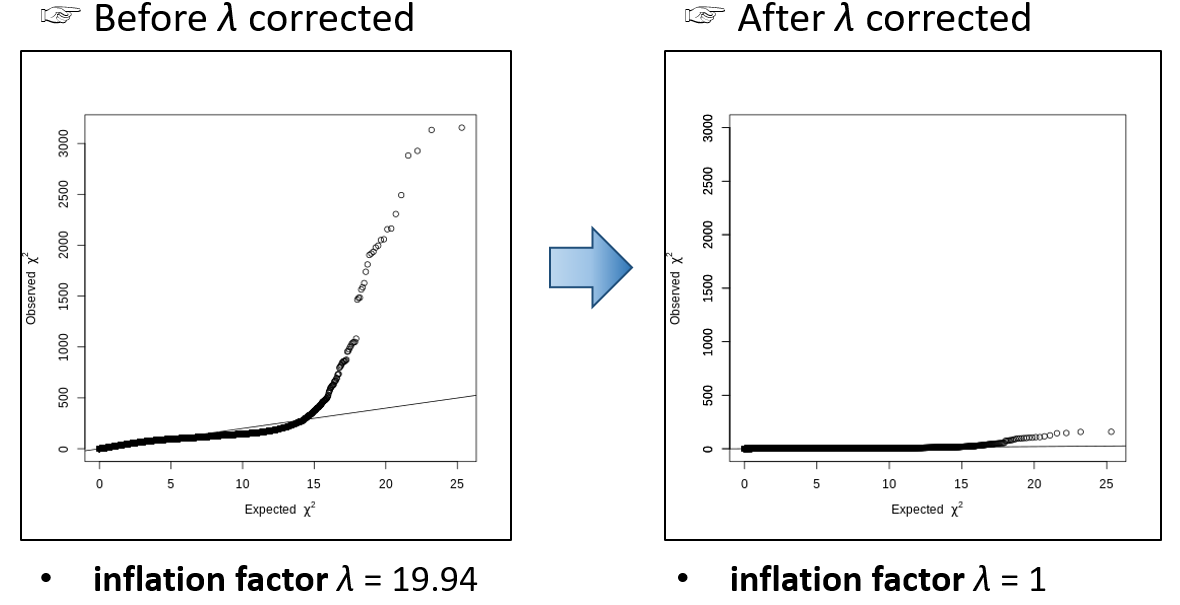

Supplement: Supplemental Information 2 — (Left) Before genomic control correction, the inflation factor (λ) was 19.94, indicating substantial test statistic inflation. (Right) After (λ) correction, the inflation factor was reduced to 1, suggesting effective control for population structure or other confounding factors. Each point represents a single SNP. The solid diagonal line represents the null hypothesis of no association. [file peerj-14-21010-s002.docx]
